# Retinal Neurodegeneration in db/db Mice at the Early Period of Diabetes

**DOI:** 10.1155/2015/757412

**Published:** 2015-03-02

**Authors:** Qin Yang, Yidan Xu, Ping Xie, Haixia Cheng, Qinglu Song, Tu Su, Songtao Yuan, Qinghuai Liu

**Affiliations:** Department of Ophthalmology, The First Affiliated Hospital of Nanjing Medical University, Nanjing, Jiangsu 210029, China

## Abstract

*Purpose.* To describe both the functional and pathological alternations in neurosensory retina in a murine model of spontaneous type 2 diabetes (db/db mouse). *Methods.* db/db (BKS/DB−/−) mice and heterozygous littermates (as control group) at various ages (12, 16, 20, 24, and 28 weeks) were inspected with pattern electroretinogram (PERG), fundus fluorescein angiography (FFA), and optical coherence tomography (OCT). Histological markers of neuroinflammation (IBA-1 and F4/80) were evaluated by immunohistochemistry. In addition, levels of retinal ganglion cell death were measured by terminal dUTP nick-end labeling (TUNEL). *Results.* Significant alternations of PERG responses and increased retinal ganglion cells (RGCs) apoptosis were observed in diabetic db/db mice for 20-week period when compared with control group. IBA-1 and F4/80 expression in microglia/macrophages became evidently for 24-week period, thus supporting the PERG findings. Furthermore, obvious thinning of nasal and dorsal retina in 28-week-old db/db mice was also revealed by OCT. No visible retinal microvascular changes were detected by FFA throughout the experiments on db/db mice. *Conclusions.* Diabetic retina underwent neurodegenerative changes in db/db mice, which happened at retinal ganglion cell layer and inner nuclear layer. But there was no obvious abnormality in retinal vasculature on db/db mice.

## 1. Introduction

Diabetic retinopathy (DR) is a leading cause of blindness among working group in developed countries [1]. It is estimated that the number of diabetes patients will achieve 366 million by 2030, the majority of which will be type 2 diabetes mellitus (T2DM). After initial diagnosis, over 80% of T2DM patients will develop DR within 20 years [2, 3]. DR is a prototypical microvascular disorder characterized by microaneurysms, capillary nonperfusion, intraretinal hemorrhages, intraretinal microvascular abnormalities, and neovascularization [4–6]. However, recent studies have identified neuroretinal abnormalities in diabetics, even before the evidence of visible microvascular changes [7–10]. DR patients usually exhibit reduced responses in full-field and multifocal electroretinography, decreased blue-yellow color sensitivity, and contrast sensitivity before the microvascular lesions occurrence [11]. Large amount of neuronal cells is detected to be damaged in the very early stage of the disease, while microvascular lesions are usually difficult to be identified by fundus photography or FFA [12], which would cause easily clinical missed diagnosis followed by worsen progression.

Retinal neurodegeneration has also been identified in diabetic rodent models, which reproduces most aspects of the early stages of DR. Lewis rats with streptozotocin-induced type 1 diabetes have shown the most accelerated loss of retinal ganglion cells (RGCs) at 8 months after the onset of diabetes [13]. The spontaneous development of diabetes in Ins2Akita mice for 5-6 months also reveals the changes in neurosensory retina, including RGCs apoptosis as well as marked alterations to the morphology of surviving cells, reduction of cholinergic and dopaminergic amacrine cells, and a distinct thinning of the inner plexiform layer and the inner nuclear layer [14–16]. Furthermore, morphologic alterations of astrocytes and microglial cells in the inner retina and impaired glutamate metabolism by Müller cells are found in other rodents [17–19]. Type 1 diabetic animal models were used in the majority of these researches, and to date retinal neural structural and functional studies of type 2 diabetic animal model are scarce.

The db/db mouse (BKS/DB−/−) is a typical model of type 2 diabetes that exhibits hyperglycemia associated with obesity a few 4–8 weeks after birth [20]. Physiological studies have revealed early inner retinal neuronal dysfunction including prolonged latencies of the oscillatory potentials and impaired b-wave at 24 weeks of age [21]. An increased apoptosis of RGCs and other retinal neurons was observed in 60 weeks old db/db mice [22]. However, the structural and functional features of inner retina on db/db mice during the early stage of diabetes remain unclear. This study was designed to follow the development of neurosensory retina damage from both structural and functional aspects in the early stages of diabetes in db/db mice.

## 2. Materials and Methods

### 2.1. Animals

Thirty-seven 8-week-old male db/db mice (BKS/DB−/−) and 37 age-matched nondiabetic littermates (BKS/DB+/+) were provided by the Animal Laboratory Center of Nanjing university and were kept in our SPF-certified vivarium and exposed to a 12-hour light-dark cycle. The mice were maintained on a standard diet and water ad libitum. All experiments conformed to standard environmental conditions (at room temperature of 23°C and humidity of 60%). To assess the chronological sequence of the retinal abnormalities associated with diabetes, 6 diabetes db/db mice were compared with 6 age-matched nondiabetic mice at difference ages (12, 16, 20, 24, and 28 weeks, resp.). Fasting blood glucose concentrations and body weights were monitored. Visual functions were measured using pattern electroretinography (PERG; Roland Consult, Germany) and in vivo retinal structures were visualized with spectral domain OCT (Cirrus HD-OCT, Zeiss Meditec, Germany). At last, mice were anesthetized with intraperitoneal injection of a mixture containing 5% urethane and 10% chloralic hydras (1 : 1) (10–20 mg kg^−1^ body weight). Whole retina samples were taken for histological examination.

All animal procedures were performed according to Guide for the Care and Use of Laboratory Animals published by the Institute for Laboratory Animal Research.

### 2.2. Pattern Electroretinogram

Detailed description of the PERG technique is reported elsewhere [23–25]. In brief, mice ensured to natural light-adapted state were weighted and anesthetized as above mentioned. Mice were fixed in a custom made holder that allowed unobstructed vision. Self-modified electrodes were made of acupuncture needles, welded to the signal-recorded wire. A recording electrode (0.25 mm diameter silver wire configured to a semicircular loop of 2 mm radius) was leaned against the extrapupillary corneal surface. Reference and ground electrodes were inserted under the skin of ipsilateral pars buccalis and tail, respectively. The pupils were not dilated, and eyes were not refracted for the viewing distance since the mouse eye has a large depth of focus. Visual stimuli consisted of horizontal bars with a contrast of 99% at 0.05 cycles/degree spatial frequency, 1 Hz temporal frequency. PERG waveforms consisted of two main waves: a positive wave with a peak latency of 90 to 100 ms (P1) followed by a broad negative with peak latency in the range of 200 to 300 ms (N1).

### 2.3. OCT Measurements

After anesthetizing, mice were dilated with tropicamide 1% and underwent spectral domain OCT (Cirrus HD-OCT, Zeiss Meditec, Germany) as reported elsewhere [26, 27]. In brief, mice were placed on the three-dimensional table. The mouse eyeball was coated with viscoelastic material and covered with a coverslip to form a plano-concave lens. Then the 90 dpt noncontact slit lamp lens (Volk, Superfield NC, Volk Optical Inc., Ohio, USA) was fixated directly in front of the OCT lens. Macular cube scanning in the cirrus HD-OCT was performed with the 512 × 128 scan pattern where a 6 × 6 mm area on the retina is scanned with 128 horizontal lines, each consisting of 512 A-scans per line with the scanning speed of 27000 A-scans per second. During the analysis procedure, the macular central fovea was aligned to the center of the optic disk. Then retinal thicknesses are averaged in nine retinal subfields which are an area of a 6 mm diameter circle centered on the mouse optic disk. The data of eight subfields except the central one was collected for statistic analysis.

### 2.4. Tissue Processing

Mice were killed at 20-, 24- and 28-week periods after visual examinations. The eyes were dipped in FFA (mixture of 95% ethyl alcohol, 10% neutral formalin, and glacial acetic acid, 17 : 2 : 1) for 1 min and fixed in 0.1 M buffer phosphate (pH 7.4) containing 4% paraformaldehyde for 2 hours at 4°C. Then, the anterior segment of the eye was dissected away, the vitreous cleared, and the eyecup was left for further fixation overnight. Eyecups were embedded in paraffin and sectioned. Serial sections 7 *μ*m thick were prepared for the assessment of retinal morphology, immunofluorescence staining, and TUNEL immunoreactivity.

### 2.5. Retinal Morphometry

After deparaffinization and rehydration, the sections were stained with hematoxylin and eosin (H&E). The measurements were taken at the regions of the retina (at 480 *μ*m form ora serrata) and were examined to ensure similar locations of measurements for all eyes. Image analyses of ten sections of each eye were used to quantify total retinal thickness, the thickness of the inner nuclear layer (INL) and outer nuclear layer (ONL). Images of H&E sections were captured with a microscope (Olympus) using the program Image J for quantification.

### 2.6. TUNEL Assay

The terminal deoxynucleotidyl transferase-mediated biotinylated UTP nick-end labeling (TUNEL) assay was performed with DAPI (4′,6-diamidino-2-phenylindole) staining [28]. The prepared tissue sections were deparaffinized in xylene for 10 min and hydrated through a graded ethanol series. The TUNEL assay was then carried out following the protocol described by the manufacturers (In Situ Cell Death Detection; Roche Molecular Biochemicals). After staining retinal sections were mounted with fluorescence mounting medium and images were obtained using an inversion fluorescence microscope (Olympus, IX2-ILL100). Quantitative observations of retinal cells count from semithin section were defined as five HP fields (40 × 10) since 200 um up from the optic papilla.

### 2.7. Immunofluorescence Staining

After deparaffinization and rehydration, the sections were incubated with ionized calcium binding adaptor molecule 1 (Iba-1; dilution 1 : 150, Wako, Neuss, Germany) and F4/80 (M-300; dilution 1 : 200, Santa Cruz) at 48°C for 2 days and then rinsed in PBS (3 × 1 hr). Then, the retina was incubated at 48°C in sheep anti-mouse and anti-rabbit antibodies conjugated to fluorescein-isothiocyanate (FITC) (Boehringer Mannheim GmbH, Germany) dilution 1 : 30 in 0.1% Triton X-100 in 0.01 M PBS at pH 7.4, for 3 days, and rinsed again. The retina was mounted on a slide in glycerol diluted with PBS at a ratio of 1 : 9. Slides were examined under a fluorescence microscope (Olympus, IX2-ILL100).

### 2.8. Data Analysis

Statistical analyses were performed using analysis of variance (ANOVA). Quantitative variables were expressed as mean ± standard deviation (M ± SE). Whole retina thicknesses obtained from all OCT devices were compared one to each other using unpaired Student's *t*-test. Statistical analyses were computerized using the STATA software (version 11.1). Levels of statistical significance were set at *P* < 0.05.

## 3. Results

### 3.1. Db/db Mice Undergo State with Obesity

**Figure 1:**
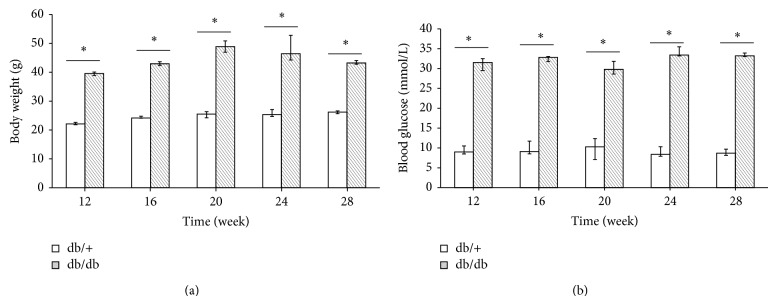
Diabetic mice (db/db) underwent a high body weight (a) and high blood glucose (b) compared to nondiabetic mice (db/+) at 12, 16, 20, 24, and 28 weeks. ^*^ *P* < 0.01.

Measurements of blood glucose (vein blood of tail using Contour TS) and body weight were conducted in diabetic and nondiabetic mice before visual function testing or they were killed for other experiments at each time point. When the result of blood glucose value was beyond the upper limit of detection (33.3 mmol/L), we set 33.3 mmol/L as the statistical default. The body weight and blood glucose values are shown in Figure 1. There were significant statistic differences of body weight and blood glucose level between the two groups, and hyperglycemia in diabetic mice runs in parallel with a significant increase of weight.

### 3.2. Altered PERG Responses in db/db Mice as Early as 20 Weeks

**Figure 2:**
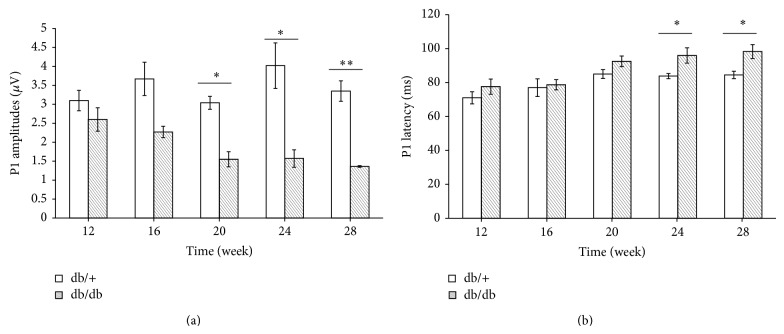
Abnormal changes in the PERG parameters of diabetic mice (db/db) were found at different week periods compared with nondiabetic mice (db/+). The P1 amplitudes and latency recorded from diabetic and control mice (*n* = 6, resp.) were compared at each endpoint. db/db mice had an insignificant lower P1 amplitude than littermates at each point and showed significant amplitude reduction for 20 weeks (a). Significant delay of P1 latency was observed in db/db mice at 24 and 28 weeks (b). Data are expressed as mean ± SE. ^*^ *P* < 0.05; ^**^ *P* < 0.01.

PERG was conducted as a surrogate measure of RGCs function. P1 amplitudes, measured from the peak of the P1 wave to the trough of the N1 wave, were slightly reduced in diabetes mice at the age of 12 and 16 weeks, but they were significantly reduced in diabetes mice when compared with controls at the age of 20, 24, and 28 weeks (Figure 2(a)). Besides, a significant delay of P1 latency recorded in the diabetic mice at the ages of 24 and 28 weeks was observed when compared with the age-matched control mice (Figure 2(b)). These data showed abnormal PERG responses in diabetic db/db mice for 20 weeks, which also suggested that RGCs dysfunction might have occurred in early period of db/db mice.

### 3.3. Retinal Morphometry Showed a Significant Retina Thinning in db/db Mice at 28 Weeks

**Figure 3:**
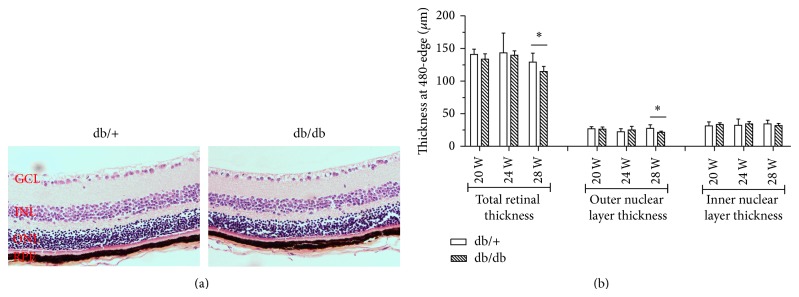
Hematoxylin and eosin stained retina (at 480 *μ*m form ora serrata) in a representative case of a diabetic mouse (upper panel) and a nondiabetic mouse (lower panel) of 28 weeks of age (a). Thickness of total retina, outer nuclear layer, and inner nuclear layer (b). Results are expressed as mean ± SE.

Measurements of retinal thickness in diabetic and in nondiabetic mice at 20, 24, and 28 weeks are shown in Figure 3. Total retinal thickness (measured from inner limiting membrane to Bruch's membrane) was significantly decreased in diabetic mice in comparison with nondiabetic mice at 28 weeks. Furthermore, a reduction in ONL was observed in diabetic mice in comparison with nondiabetic mice at 28 weeks.

### 3.4. OCT Showed a Significant Retina Thinning in db/db Mice at 28 Weeks

The retinal thickness was measured by OCT noninvasively in 7 diabetic db/db mice and 7 nondiabetic mice at 28 weeks. One eye was selected at random in each mouse and only that eye was included in the analysis. As shown in Table 1, OCT revealed a significant thinner in total retinal thickness of db/db mice than the control mice (*P* < 0.01). Specifically, the differences were significant also for all quadrants. Moreover, both the inner and outer sectors thicknesses of superior as well as nasal quadrants in db/db mice were significantly thinner than nondiabetic mice. Additionally, significant thinning occurred in inner sector of the temporal quadrant in diabetic mice compared with control mice, despite the fact that no significant difference was detected in the temporal quadrant between the two groups.

### 3.5. No Apparently Visible Microvascular Abnormalities Observed by FFA

**Figure 4:**
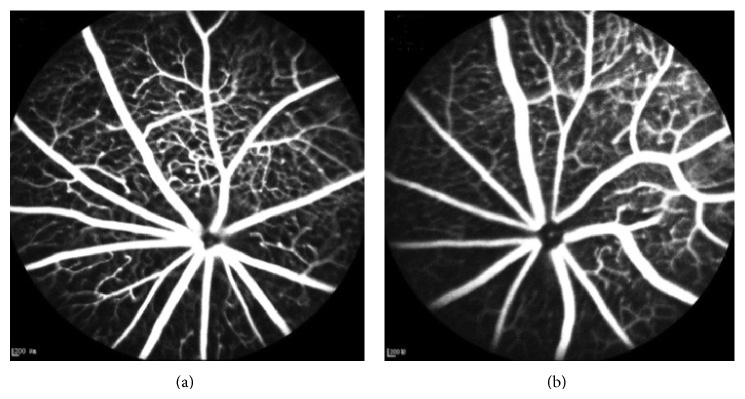
No evident retinal vascular changes were observed in db/db mice by FFA. FFA image in db/db mice (a) and db/+ mice (b) at 28 weeks. Neither of them presented abnormal alterations on retinal vascular structures.

After examinations of PERG, FFA were conducted which monitor the flow of a fluorescent dye through the retinal vasculature. All db/db as well as control mice did not show any alternations corresponding to relevant vascular abnormalities at each endpoint.  Figure 4 shows the images of FFA from db/db and db/+ mice at 28 weeks and there was no visible neovascularization and cellular capillaries in the retina.

### 3.6. Cells Apoptosis Found in RGCs Layer and the Inner Nuclear Layer

**Figure 5:**
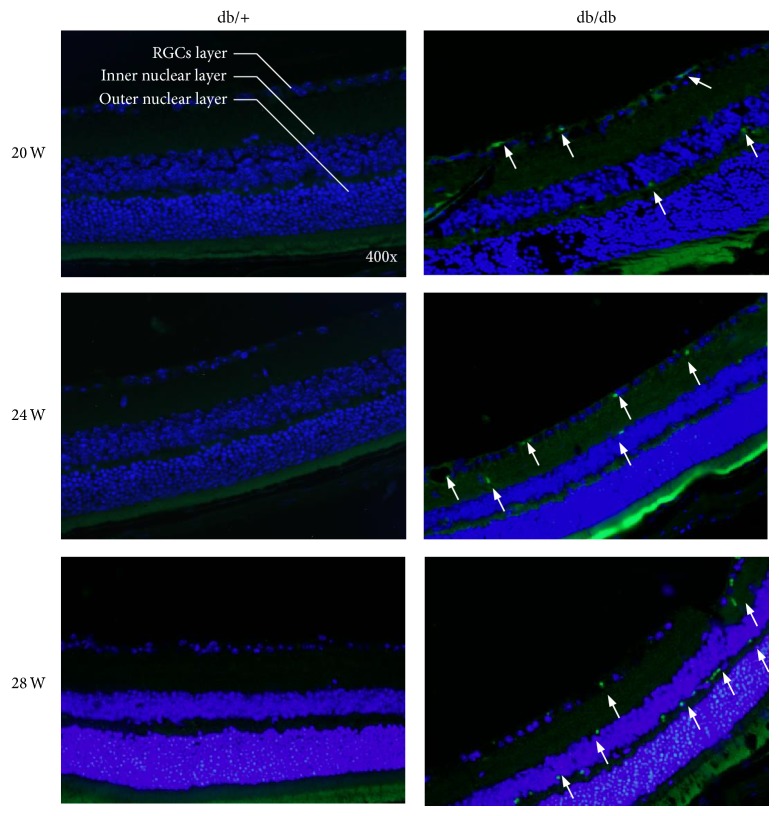
TUNEL assay showed the positive apoptotic RGCs in retina of db/db mice at 20, 24, and 28 weeks of age compared with the controls at 28 weeks. The green fluorescence represented the apoptotic cells in RGCs layer and the inner nuclear layer which were noted by the arrows.

Retinal sections were stained for dying photoreceptors by TUNEL staining to further determine the histological retina changes. A significant increase in TUNEL-positive immunofluorescence was observed in diabetic mice in comparison with retinas from nondiabetic mice at 20, 24, and 28 weeks (Figure 5). TUNEL-positive cells were mainly localized in the RGCs layer, as well as the INL, implying the RGCs apoptosis which was also suggested by PREG results. In addition, the number of apoptosis cells was increased from 1-2/HP at 20 weeks to 8-9/HP at 28 weeks (Figure 5).

### 3.7. Possible Neuroinflammation Was Observed in Early Periods of db/db Mice

**Figure 6:**
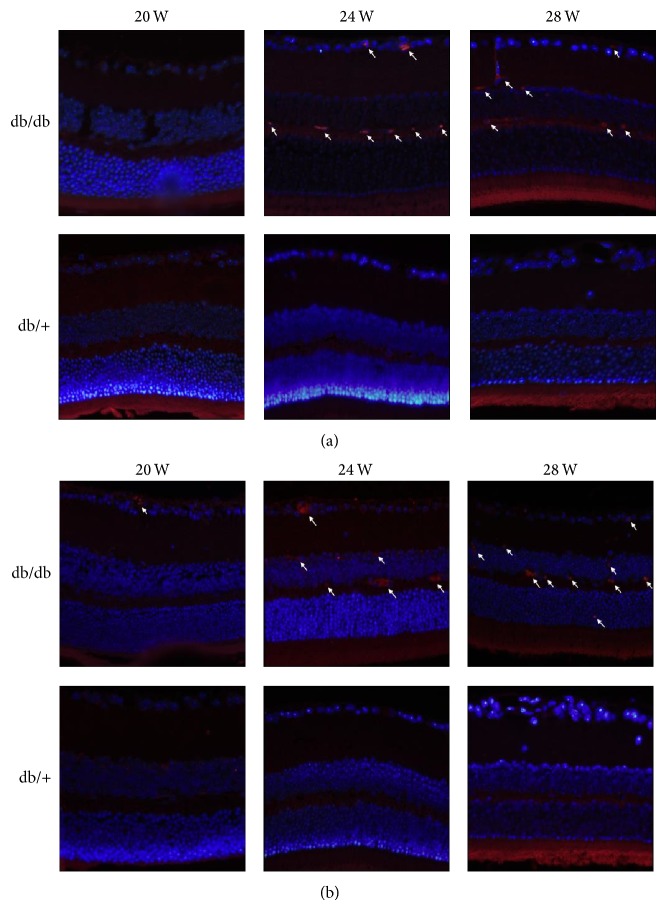
Effect of diabetic condition suffered the db/db mice on inflammoactivation in the retina. Representative immunofluorescence of Iba-1 ((a), 400x; microglia, macrophages) and F4/80 ((b), 400x; microglia, macrophages) was observed in db/db mice eyes at 24- and 28-week period compared with db/+ mice. No positive expression of the two markers was found in littermates eyes. Arrow represented the positive expression.

To confirm the possible neuroinflammation in retinal neurodegeneration, Iba-1-labeled microglia/macrophages and F4/80-labeled mature macrophages staining were performed. As shown in Figure 6, no positive expression of the two markers was observed in either 20-week-old diabetics or nondiabetics mice. However, db/db mice at 24 and 28 weeks showed obvious fluorescence of positive IBA-1 and F4/80 expression in RGCs layer and INL, while no specific immunofluorescence exhibited in the retina of control mice (Figure 6(a)). Moreover, positive F4/80 was also expressed in the ONL of db/db mice (Figure 6(b)). These results confirmed the presence of IBA-1 and F4/80 positive cells in RGCs layer and INL by the time of 24 weeks.

## 4. Discussion

When compared with normal heterozygous littermates, the db/db (Lepr^db^) mice can be affected with hyperglycemia, hyperlipidemia, and accelerated degeneration of the retinal capillaries and pericytes [21]. It has been reported that 26-week-old db/db mice showed early features of DR, manifesting pericyte and endothelial cell loss, Bruch's membrane thickening, and increased blood flow in the retina [29–31]. However, rodent models can reproduce most aspects of the early stages of DR but fail to develop retinal neovascularization or other advanced lesions (retinal hemorrhages and microaneurysms) of retinopathy [32]. Although Cheung et al. [22] had reported that 15-month-old db/db mice showed increased density of retinal capillaries in the inner nuclear layer, in current study, we did not found any obvious microvascular changes using FFA in db/db mice alive for 28 weeks. Moreover, the reduction of retinal thickness in the db/db mice by using OCT also supports that there was no retinal edema and/or retinal blood barrier damage. The relatively short lifespan of the mice and thus shorter duration of diabetes (most studies are <1 year) were probable reason for failing to observe neovascularization in these models. Therefore, the results of our study were supporting previous researches which implied that db/db mice developed capillary changes under microscope even in the early period of life span, but the lesion was not severe enough to cause the breakdown of retinal blood barrier.

The results of PERG presented in our study indicated that diabetic condition acts to be injurious events that lead to exacerbation of RGCs death. PERG signal depends on the structural and functional integrity of RGCs and will greatly reduce when RGCs were selectively degenerated after optic nerve lesion [33]. PERG, therefore, is extensively used to probe RGC function in clinical and experimental models of diabetes and optic neuropathies [34–36] and may be more sensitive to assess early damage of DR.

Previous studies showed that PERG impairment could be detected in diabetic patients without or with minimal signs of retinopathy. This suggests that retina functional loss may occur in the early stage of this disease. Therefore, we used PERG to evaluate the changes in RGCs function and we found that the P1 amplitudes significantly reduced at the follow-up examinations for 20 weeks in db/db mice; then a significantly increased latency was found later. All these results indicated that the deficit function of RGCs in db/db mice had certainly occurred.

It was reported that RGCs die in a compartmentalized manner such that the mechanisms responsible for death of the RGCs body and dendrites may be different from those responsible for axonal injury [37]; both of them compromised the RGCs dysfunction. OCT was performed as an indicator of progressive neural retinal pathology in animal models of retinal degeneration [38, 39]. In the present study, we found a significant reduction in overall retinal thickness as well as all four quadrants, depending on the OCT scanning, in db/db mice when compared with control mice at the age of 28 weeks. Moreover, the results of H&E showed that total retinal thickness as well as ONL (at 480 *μ*m form ora serrata) was significantly decreased in diabetic mice in comparison with nondiabetic mice at only 28 weeks, but there was no difference between the 2 groups at 20 and 24 weeks. These results are mainly due to the apoptosis of the ganglion cell layer and photoreceptors [40]. In a recent study, Bogdanov et al. [40] reported that total retinal thickness in both central and peripheral retina was significantly decreased in db/db diabetic mice at 16 and 24 weeks, and a thinning in both ONL and INL as well as a reduction in the number of cells in the GCL was observed at 8, 16, and 24 weeks. Unfortunately, in our study only the db/db mice at 28 weeks were measured by OCT, with the original intention to validate the reduced PERG response. Whether there existed OCT alternations before 28 weeks is still unclear. So further studies are needed to determine the early changes of OCT in db/db mice and evaluate the correlation between OCT measured and histologically measured changes in retinal thickness following the life periods.

Recent studies have approached a common view that cell apoptosis is the base of diabetic neurodegeneration in cytopathogenesis [41]. Reportedly, various retinal neurocytes in early DR have been in apoptosis state, in particular the RGCs [42–44]. Accompanying cell apoptosis, inflammation has been considered to be an important pathogenesis in early stages of experimental DR, which has shown both of microglial and macrophages reaction represented during the alternations of functional and structural in retina [45–47]. Here, to further confirm the RGCs dysfunction determined by PERG, TUNEL assay was performed to detect the cell apoptosis in retina. Previous study reported that 15-month-old db/db mice had increased apoptosis of RGCs and other cells in the neural retina [8]. In our study, apoptotic RGCs were observed in 20-week-old db/db mice, consistent with the reduced PERG amplitudes. Moreover, the number of apoptotic cells was increasing with age, accompanied with evident induction of IBA-1 and F4/80 expression, both of which were considered as established marker for microglia/macrophages in the retina [48]. Additionally, IBA-1 and F4/80 positive cells as well as apoptosis cells were also found in the INL, which was implied to be affected by db/db mice. All these findings indicated that db/db mice undergoing diabetes in 20 weeks or longer had developed neurodegeneration and neuroinflammation.

In conclusion, we demonstrated that functional and structural retinal neurodegeneration exactly occurred in db/db mice with type 2 diabetes mellitus as early as 20 weeks, preceding any detectable alternations of blood vessel visibly. The retina lesions that develop in type 2 diabetes are similar to those that develop in type 1 diabetes, although the severity and/or incidence may be different [49]. Thus, it is prominently important to trace the degenerated phases and change rule of diabetic nerve cells in the early stage of DR and timely block and reverse these cells' degeneration. These actions would play a significant role in prevention of early diabetic retinal neuropathy and nerve protection treatment, which are promising to be a potently effective target for the early intervention of diabetic retinopathy in future.

## Figures and Tables

**Table 1:** Retinal thickness from OCT in diabetic and nondiabetic mice at 28 weeks.

Thickness (*μ*m)	Db/db mice	Db/+ mice	95% confidence interval	*P* value^#^
Global retina	221.7 ± 2.27	237.48 ± 1.98	[−22.33, −9.24]	0.0002^**^
Superior	214.36 ± 2.26	230.21 ± 2.68	[−23.49, −8.23]	0.0007^**^
Nasal	224.21 ± 3.09	238.93 ± 1.28	[−22, −7.43]	0.0009^**^
Inferior	230.86 ± 4.15	245.43 ± 2.25	[−24.85, −4.29]	0.0094^**^
Temporal	217.36 ± 1.82	235.36 ± 2.6	[−24.91, −11.09]	0.0001^**^
central	179.57 ± 5.92	196.29 ± 10.16	[−42.32, 8.9]	0.1805
Superior-inner	204.86 ± 3.32	223.71 ± 4.21	[−30.53, −7.18]	0.0042^**^
Superior-outer	223.86 ± 3.59	236.71 ± 1.6	[−21.42, −4.3]	0.0067^**^
Nasal-inner	218.29 ± 3.3	234.86 ± 2.35	[−25.4, −7.74]	0.0015^**^
Nasal-outer	230.14 ± 3.15	243 ± 1.63	—	0.0103^*^
Inferior-inner	228.43 ± 3.48	244 ± 2.69	[−25.16, −5.98]	0.0041^**^
Inferior-outer	233.29 ± 6.18	246.86 ± 3.03	[−28.59, 1.43]	0.0723
Temporal-inner	205.43 ± 2.38	227 ± 3.69	[−31.14, −12]	0.0004^**^
Temporal-outer	229.29 ± 2.96	243.71 ± 1.94	[−22.13, −6.73]	0.0015^**^

^#^Retinal thicknesses in db/db mice and littermates were shown as mean ± SE, *n* = 7 in each group, and one eye was selected at random in each mouse and only that eye was included in the analysis; unpaired student's *t*-test was used to analyze the thickness data. Wilcoxon rank sum test was used in the analysis of nasal-outer thickness due to the sample skewed distribution. The central fovea thickness was not included in the whole thickness of global retina. ^*^ *P* < 0.05. ^**^ *P* < 0.01.
